# Stressors and starting points for health-promoting interventions in medical school from the students’ perspective: a qualitative study

**DOI:** 10.1007/s40037-015-0189-5

**Published:** 2015-06-02

**Authors:** Thomas Kötter, Nadine J. Pohontsch, Edgar Voltmer

**Affiliations:** 1 Institute for Social Medicine and Epidemiology, University of Lübeck, Ratzeburger Allee 160, 23552 Lübeck, Germany; 2 Department of Primary Medical Care, University Medical Center Hamburg-Eppendorf, Hamburg, Germany; 3 Chair of Health Sciences, Friedensau Adventist University, Möckern, Germany

**Keywords:** Education, Medical, Stress, Psychosocial, Health promotion, Focus groups

## Abstract

**Introduction:**

Medical education is considered very challenging and connected with high levels of psychosocial stress for students. The aim of this study was to identify stressors and possible starting points for health-promoting interventions from the perspective of the students themselves.

**Methods:**

We conducted two focus groups with medical students from pre-clinical and clinical semesters. We analyzed the data using content analysis following Mayring’s approach.

**Results:**

The stressors in the pre-clinical stage of medical education were more diverse and perceived as more intense than those in the clinical stage. They comprised contextual factors and individual behaviour. Participants mentioned the weekly examinations as a specific stressor. The existing absence regulations gave the participants the impression that they should not be absent through illness at any point during the course, and this idea further promoted presenteeism. Peer groups and mentoring programmes were perceived as helpful.

**Conclusions:**

Stressors and starting points for health-promoting interventions are closely related to the medical curriculum and its organization. As such, the curriculum itself—in addition to programmes aimed at improving stress management—should primarily stand at the centre of activities for enhancing students’ health.

## Introduction

Numerous studies indicate that medical students are exposed to a number of stressors that negatively affect their psychosocial health during the course of their studies [1–3]. At the end of medical education [3], and after the internship year [4], about 20 % of students show signs of burnout, which is associated with low empathy [4]. Comparative studies with students from other disciplines show that medical students have a stronger commitment [5] and higher levels of perceived stress [6]. The authors of a Swiss study found that medical students were among those with the highest psychosocial stress values and the least social support, correlates of an increased risk of physical and mental diseases [7]. Bernhardt et al. studied distress associated with dissection during the anatomy course, which may be regarded as a specific stressor for medical education, and found increased values for about 50 % of the students [8]. The need for interventions to promote the health and resilience of students is evident, which is also true in the perception of medical students themselves [9].

To date, however, literature on the implementation of health-promoting interventions in medical schools in Germany has been limited (e.g., Jurkat et al. [10]). Internationally, different approaches to health-promotion interventions in medical schools have been described [11–13], but the efficacy has not been evaluated using high-quality study designs [14]. Evidence for selecting appropriate interventions is therefore currently limited. A common suggestion for the development of health-promoting interventions is to involve those who are affected by and who are experts in the situation at an early stage [15]. As a first step, we were therefore interested in evaluating the following, using a qualitative design:

- Which medical school-related stressors do the students perceive as relevant?
- Which health-promoting interventions might be suitable to either reduce exposure to these stressors or to facilitate dealing with them from the students’ point of view?

## Methods

### Study design

To explore perceived medical school-related stressors, as well as starting points for health-promoting interventions, we conducted two focus group discussions.

### Participant selection

In order to interview students knowledgeable on the topic, we invited all students who had attended the elective ‘Health and Well-being for Medical Students and Physicians’ during their pre-clinical studies at the University of Lübeck to participate. As an incentive, we offered a reward in terms of a book voucher to the amount of 5 €. A total of 16 students (8 female and 8 male) participated in two focus groups. Of these, 10 participants were in the pre-clinical stage of study (4/8 in one focus group and 6/8 in the other).

### Setting

The study centre was the University of Lübeck, a public university with a focus on medicine and life sciences.

### Data collection

The focus groups were moderated by two scientists (TK: post-doctoral research fellow and physician; EV: professor of health sciences and physician) using a semi-structured guideline [16], logged by an assistant and recorded digitally. Due to a technical problem, only the second focus group was transcribed in full. For the first focus group, therefore, the detailed field notes of the meeting were included in the analysis. The participants were informed about the fact that the session was recorded and that the data would be analyzed anonymously for scientific purposes. No one decided to withdraw from participation. At the beginning of the discussion, the students were asked to name and discuss medical school-related stressors. The stressors mentioned were recorded on a flip chart. Moderated by TK and EV, students were asked to suggest and discuss solutions to cope with and reduce medical school-associated stress. Proposals were recorded on paper cards and pinned to a board. Finally, the students were asked to prioritize the suggestions for health-promoting interventions by labelling the three most important proposals using adhesive dots. Proposals were then ranked according to their scores and discussed.

### Data analysis

The interviews were analyzed using qualitative content analysis. This systematic procedure is used to reduce large amounts of data while preserving and extracting the main content [17]. The manuscript and minutes of the focus groups were read several times before coding to ensure familiarity with the material. Deductive categories were derived from the aims of the study and the focus group guideline. Due to the exploratory nature of the study the focus was on inductive coding during the review of the material. The data were coded independently by two investigators (TK and EV). Discrepancies in coding were discussed and resolved, if necessary, with the help of another investigator (NP: post-doctoral research fellow and psychologist). Two of the authors (TK and EV) are medical school graduates, and thus bring an insider perspective to the work. In order to ensure a balanced perspective, the coding system was discussed in depth with the third author, who is not a medical doctor and was not involved in the data collection. Afterwards, all material was coded again using the final category system. The data analyses were conducted using MAXQDA, version 10.

### Ethics

The study protocol was approved by the Ethics Committee of the University of Lübeck (file reference: 11–010).

## Results

The results of the qualitative evaluation are divided into two sections: (1) Perceived stressors, and (2) Starting points for health-promoting interventions. In the following sections, each theme is discussed with relevant quotations (for an overview of the code system, see Table 1).

**Table 1:** Stressors and starting points for health-promotion interventions (code system)

Stressors	Starting points for health-promotion interventions
**Level of individual behaviour and experience**	
Expectations of others	Exchanges with more advanced semesters
Costs	Creating awareness/sensitization
Social conflicts	Nutrition
Responsibility	Time management
Nutrition	Relaxation
Fear of malpractice	Social support
Being alone	Leisure/compensating activities
Competition	Absence regulation
Overloading yourself	Learning
Perfectionism	Attitude
Procrastination	
Lack of orientation/overview	
Internal motivation	
**Setting level**	
Cafeteria	Counselling centre
Library	Curriculum
Curriculum	Nursing internship/clerkships/PJ
Break times	Teaching evaluation
Wealth of curricular material	Marks
Quality of teaching	Break times
Experienced futility	Subjects
Grading	Medical Psychology
Lack of consistency of evaluation	Career Exploration
Lack of clinical relevance	Short lecture on physicians health
Heteronomy	Biochemistry
Doctoral thesis	Documents
Nursing internship	Laboratory
Tests	Quality of documentation
Exams	Examinations
Oral attestations	Ethics
Continuous load	Organization
Tests at the end of the semester	Implementation
Stressful	Prioritization of the learning content according to exam and practice relevance
First medical exam	Cafeteria
Absence regulations	
Seminars and laboratories	
Physiology	
Ethics	
Microbiology	
Pharmacology	
Double load	
Overlays	
Medical psychology	
Biochemistry	
Additional seminar	
Quality of documentation	
Examinations	
Didactics	

### Stressors

#### Examinations

As important stressors in the pre-clinical stage, students mentioned factors attributable to the curriculum. At first, the weekly examination rhythm in the main subjects-Anatomy, Biochemistry and Physiology-was criticized. The workload was perceived differently in relation to the different subjects. *‘Before Anatomy, I almost always cried’*; *‘So, yes, I found the [Anatomy] intermediate examinations harder, but they had a meaning. I find intermediate examinations in Physiology and Biochemistry just pure harassment because we’ll have to pass the final examinations anyway. And then I ask myself: Why do we have to do this?*-*The grades awarded have no consequences, except in that they somehow induce stress’*.

This perceived pressure was morally elevated by pronouncements from teaching staff, such as: ‘*One cannot actually afford, as a medical student, to not know something, because one will finally treat people and could potentially kill them through ignorance!*’

In contrast, however, there was a clear sense that these moral elevations could possibly be misguided *(‘But, after all, one must somehow stay human and be allowed to err from time to time’*).

Overall, the meaning of the escalating pressure was questioned (*‘Actually, you only need 60 % to pass the exam’*) and it was suspected that the well-being of students is less important for the teaching staff than the standing of their institution in terms of overall performance *(‘It is the objective of the Biochemistry staff to constantly score first place in the overall examination results’*).

#### Absence

Very long laboratory hours were frequently mentioned *(‘But the laboratories are shorter [in the clinical stage] so they do not last until late. In Biochemistry, one has to stand for 7 or 8 h’*). Students felt that they were not allowed to be absent, even when sick *(‘I find it somehow unacceptable that in some subjects you have absolutely no allowed absences and in others only 1 or 2 days’; ‘Then, due to one lousy day of absence, you have to add half a year to your course!’*).

Such practice in relation to absence was not only perceived as negative in terms of the courses but also for internships.

The ironic statement ‘*Medical education should prepare students in advance for the reality that as a doctor one is not allowed to be sick because one must heal the sick. One is somehow no longer a human being*’ implies that this strict absence regime is perceived as contradictory to their own professional understanding.

#### Internal motivation

In addition to external factors, the students are also aware of a strong internal motivation: *‘Every time I go into this laboratory, I think: It’s just a laboratory, it is not your life. It is of micro-minimal importance in relation to your whole life. And then, when I’m standing there, I realize at some point that I am causing myself too much stress, and I think: This test tube is your life at the moment and you have to give everything. It just builds up such pressure!*’

Especially in the pre-clinical stage, students lack advice regarding which courses are indispensable and which are more or less optional: *‘During the first semester, I learned Anatomy until complete exhaustion late in the evening. And I really made myself ill because no one said to me: ‘that’s enough now’, or ‘you may learn this and that a little more superficially’.*

#### Lack of prioritization and clinical relevance

Catalogues for learning objectives as an aid to prioritization of learning content and to estimate relevance for examinations are missed: ‘*I perceived this as the worst problem in Biochemistry, whereas I also sometimes had the feeling in Pharmacology*-*that it was exactly the same thing. They have an exact catalogue of learning objectives but then you sit in the exam and they ask something wacky, something no one cares about anyway and that no one needs to know except pharmacology students. Why have I written and learned stacks of notes only to be confronted with that sort of stuff!*’

Even during the clinical stage, students feel that many exotic diseases and content are prioritized over what is considered relevant for future clinical practice and everyday life: ‘*If I find at the end of a textbook chapter the sentence ‘this disease is very rare’, I feel frustrated.*’

#### First medical exam

The first medical exam was named as a significant stressor during pre-clinical medical education. Statements made by teaching staff which are meant to offer relief to students are perceived as a burden by them: ‘*[…] I find the being told that 95 % of students pass the course unhelpful because that means that 5 % do not pass. And I simply think, if you are one of those 5 % then you feel like the greatest deadbeat in the world because actually the vast majority of students pass*’.

Compared with the pre-clinical section, the spectrum of stressors is different in the clinical stage. Subjects requiring extensive learning, such as Microbiology and Pharmacology, are still mentioned, but these are perceived as less burdensome and clinically relevant (‘*Then it becomes more and more practical. You cannot actually compare the clinical and pre-clinical stages*’).

#### Dissertation and medical responsibility

New challenges in the clinical section are the parallel work on a doctoral thesis and a growing feeling of responsibility in patient care (‘*So I’m in my tenth semester. What now stresses me is the thought that it’s now nearing the time when I have to take care of patients on my own. That leads me to worrying about the responsibility rather than focusing on how I pass the exam*’). Overall, there is a decreasing feeling of being part of a group and a stronger impression of being alone in the clinical stage.

### Starting points for health-promoting interventions

Starting points for health-promoting interventions mentioned by the participants can also be assigned to the levels of the setting and individual behaviour and experience. Naturally, these starting points are closely associated with the aforementioned stressors, but they do not resemble them completely (Table 1).

#### Prioritization of educational content

The most frequently mentioned starting points for health-promoting interventions on the setting level were related to the curriculum itself. Given the wealth of learning content, participants wished that there was a prioritization of this content and proposed the creation of learning objective catalogues, especially in the most extensive pre-clinical subjects such as Biochemistry and Anatomy, as a particularly suitable starting point.

A clearer distinction between vital basic knowledge that must be mastered by all and additional, more facultative knowledge seemed desirable to the students. The question of clinical relevance was asked particularly with regard to the basic science subjects (‘*Most of the content you have to learn for the first medical examination, you will never need again*’). Another important criterion for the prioritization of learning content was the relevance for examinations (‘*I wonder if it is really necessary for us to do a laboratory because the content is not relevant for the exam*’).

#### Redistribution of workload

A schedule that does not contain learning-intensive subjects, e.g. Biochemistry and Anatomy, at the same time in the semester course is a further starting point for a health-promoting measure suggested by the students.

#### Grading system

The need and relevance for the grading of course performance was criticized: ‘*That’s right, you could actually omit these grades in Biochemistry because it really only exerts pressure*’.

#### More flexible absence rules

The change in policy regarding absence appeared to be very important for the students: ‘*In the end, you should tell your patients: Stay home if you’re sick. And what kind of role model are you if you are not doing it yourself?*’

#### Curricular offers for study organization and health promotion

Promoting exchanges with students from advanced semesters, for example as part of (peer) mentoring groups, was proposed by the students as a starting point at the individual level (‘*In the first semester, my peer mentors told me which lectures were and were not worth attending. If I’d attended all the lectures, I wouldn’t have passed the exams*’). The participants also identified as useful the facilitation of compensatory activities, such as sports and relaxation. Skills such as ‘learning to learn’ or time management were also considered starting points for health-promoting interventions.

The elective course ‘Health and Well-being of Medical Students and Physicians’ was rated positively: ‘*The project of personal health promotion in the elective was very helpful. The permission given and the fostering of the ability to not constantly have to learn, but also to take care of your own health, were most useful*’.

#### Further starting points

Approaches such as an improvement in the quality of cafeteria food and a low-threshold counselling service for students were suggested.

#### Final prioritization

The final prioritization of proposals for health-promoting interventions resulted in a similar picture from the two focus groups. In both cases, principal issues among the setting-based interventions were:

- prioritization of learning material,
- formulation of learning objectives,
- availability of psychosocial counselling and
- awareness of the issue of students’ health.

One of the two groups advocated:

- a reform of the strict absence rules,

and placed this first on the ranking of possible actions.

At the level of individual behaviour:

- exchanges with individuals from more advanced semesters, for example in the context of a mentoring programme,

was considered an important measure. Students felt that this would allow them to learn from older medical students about the possibilities for prioritization of learning material in order to be able to issue oneself ‘permission for leisure’.

## Discussion

By means of two focus groups with medical students we evaluated stressors and starting points for health-promoting interventions in medical school. The most important stressors, and the suggested measures, were related to the curriculum itself and the organization of the course of study.

### Stress management

Not only discipline-specific but also comprehensive issues, such as the volume of learning content, the dense schedule, examination characteristics and absence regulations seem to have a high potential to impair students’ health [1]. Students are aware of what Lazarus and Folkman described in the transactional stress model: internal and external factors work together [18]. While some students experience the vast amount of learning content as a challenge without distress others experience anxiety and a feeling of threat.

This is consistent with other study results. Aster-Schenck et al. [9] asked medical students in a quantitative study about preferences for preventive measures in their curriculum. Most of the students in this study wished for measures for stress and time management, and the prevention of burnout. Setting-related measures were not included in this study. Likewise, at the level of the individual, measures of stress management were most frequently favoured as health-promoting measures in a health survey of Bielefeld students. At the setting level these included an extension of non-smoking areas, a better choice for healthy Mensa food, restrictions in the selling of alcohol and better medical supplies [19]. In a description of the development of a health-promoting university in the UK [20], the provision of psychosocial counselling is mentioned (alongside other points) as an important issue. At the individual level there were also topics consistent with our results: conveyance of stress management techniques, improved access to a healthy diet and related counselling, fostering of exercise and other components of a healthy lifestyle.

Support for the management of study-associated stress seems to be an issue consistently mentioned by students in respective studies.

### Critical citing of the wealth of curricular material and early relation to practice

The wealth of material in medical education has been an issue of critical discussion for years. A revision of the study content with the goal of securing the opportunity to study while in good health seems to be much more meaningful [21] than helping to make students fit enough to withstand the stress of medical school. Such an approach might also foster a learning by interest and not-which has been seen in the results-driven by the relevance of content for the examination. This would also be more in line with the declaration of the World Health Association for health promotion (Ottawa Charta 1986) [15], in which healthy settings for work and living are claimed.

In addition, the feeling of a lack of clinical-related practice, especially in the pre-clinical section, has to be noted. A number of Anglo-American studies have shown that early clinical experiences not only increase students’ motivation to study and their security and competence in handling patients [22–24], but also foster their readiness to study basic sciences [25, 26]. Not least these insights led to the reformation of medical education in nine German Universities, all of which now practice a strong integration of basic science and clinical application from the beginning of the course. The reported lesser strain in clinical years is also indicative that both sections should be more integrated. Increasing the amount of patient contact may also help to overcome the insecurities of students in more advanced semesters in terms of practical handling requirements when starting professional life. In an evaluation of the reformed courses of study, the German Council of Science and Humanities recommends cancelling the classical split of pre-clinic and clinic in favour of a comprehensive integration of the theoretical and clinical study content. Moreover, the wealth of material should be reduced to a core curriculum with opportunities to set individual emphases [27].

### Grading systems and absence rules

Grading *in practicum* in addition to extensive and also graded written exams at the end of the semester was perceived as stressful. Reed et al.’s results show for the US that compared with differentiated marks, a pass/fail grading causes much less stress, less emotional exhaustion and depersonalization, a lower risk for burnout and a lower probability of quitting the course of study [28].

Another problematic issue for the students was the regulation for absence. Independent of the reason, a missed day in certain laboratories can only be made up for in the following semester. According to the students, this fosters a culture of ‘presenteeism’, which means showing up at teaching events or work even if you are ill [29]. ‘Presenteeism’ seems to be common among physicians, not only in Germany [30], and may be rooted in experiences during medical education. Institutional regulations that promote student health by explicitly discouraging ‘presenteeism’ are required.

### Psychosocial counselling and support

Since a psychosocial counselling address for students of this university already exists, it is surprising that the students demand further counselling service. A personal talk with the psychologist in charge revealed that for many years the demand has exceeded the supply. Improvement is planned. This statement is consistent with the results of a nationwide survey of workers in council offices of the German Student Services Organizations [unpublished work]: More than half of these experts rated the personal capacities of the council offices as totally inadequate. In addition, our results reveal that the visibility of these institutions should be fostered.

### Strength and weaknesses

The early and comprehensive participation of those affected in the development of health-promoting interventions is a general demand of the literature [20]. Given the scarcity of respective literature, this can be seen as a strength of our study. Our participants were not representative of the whole student population (all were knowledgeable on the topic by having completed the elective, and women and students from the clinical stage were underrepresented when compared to the whole student population). However, representativeness was not intended in the first place (see *Methods*) for this initial qualitative evaluation. It will, of course, be of importance for quantitative studies in the future (see *Implications for practice and research*).

The lack of a complete transcript of the first focus group was compensated for by a differentiated protocol and the documentation on the flip chart.

### Implications for practice and research

In contrast to health-promoting concepts and activities that focus on the individual, our results emphasize a shift towards setting-based approaches. Changes in the organization of the study, including more tolerable scheduling and more flexible absence rules, should be developed in participation with those affected by or responsible for the measures. In addition, the implementation of interventions that train students to be aware of and to cope with stress, should be fostered within the curriculum at medical faculties.

Representative quantitative research should be completed in relation to our qualitative results. These surveys should also include students from other disciplines to reveal commonalities and differences.

### Conclusions

The qualitative survey of stressors and suggestions for health-promoting interventions revealed that problems and solutions were mainly seen at the level of the setting, primarily the organization of the course of study, and to a lesser extent at the level of the individual student. Aspects related to the curriculum, such as the wealth of material, the absence of hints for prioritization and absence rules that foster ‘presenteeism’, were perceived as risk factors for health.

Important measures for health promotion included a prioritization of learning content by academic staff and students from more advanced semesters as well as an extension of capacities for psychosocial support.

## Essentials

- Medical students clearly identified stressful experiences in their education.
- These stressors are mainly curriculum-associated.
- The pre-clinical stage appears to be extraordinarily stressful.
- Changes in the curriculum itself appear to promise starting points for health-promoting interventions.
- Health-promoting interventions can help students to reduce avoidable stress and to cope with unavoidable stress.
